# Photobiomodulation for primary wound healing and patient-reported outcomes after esthetic crown lengthening surgery: A pilot randomized clinical trial

**DOI:** 10.1007/s10103-026-04950-0

**Published:** 2026-07-20

**Authors:** Marcela Letícia da Silva Azevedo, Francisco Leonardo da Silva Júnior, Tasso Ehm Martins, Ádylla Rominne Lima Barbosa, Valkleidson Santos de Araújo, Régia Carla Medeiros da Silva, Mariana Linhares Almeida-Veloso, Euler Maciel Dantas, Bruno Cesar de Vasconcelos Gurgel, Ana Rafaela Luz de Aquino Martins

**Affiliations:** 1 Dentistry Departament, Federal University of Rio Grande do Norte, Natal, Brazil; 2 Faculdade de Enfermagem Nova Esperança, Mossoró, Brazil

**Keywords:** Low-level laser, Photobiomodulation, Biostimulation, Dental esthetics, Gummy smile

## Abstract

To evaluate the efficacy of photobiomodulation (PBM) on primary wound healing and patient-centered outcomes following esthetic clinical crown lengthening with osteotomy over a three-month follow-up period. Twenty-seven systemically healthy patients presenting < 10% bleeding on probing and altered passive eruption were enrolled in a split-mouth, triple-blind, randomized controlled clinical trial. Each patient underwent esthetic crown lengthening surgery in two hemi-arches: a control group (CG), treated with surgery alone, and a test group (TG), treated with surgery followed by PBM (660 nm, 100 mW, 30 J, 5 min, sweeping motion). Laser irradiation was performed immediately after surgery and on postoperative days 4, 7, 11, and 14. Clinical assessments were conducted at baseline and at 4, 7, 11, 14, 45 days, and three months postoperatively. Outcomes included bleeding on probing, probing depth, gingival phenotype, width of keratinized mucosa, wound healing assessed by the modified Landry Healing Index, postoperative pain measured by a numerical visual scale, oral health–related quality of life (OHIP-14), patient satisfaction, and esthetic outcomes assessed by the Pink Esthetic Score. Statistical analyses were performed with a significance level set at *p* < 0.05. All patients completed the study and remained periodontally healthy throughout the follow-up period. No statistically significant intergroup differences were observed for wound healing, postoperative pain, or professional esthetic outcomes at any evaluation time point (*p* > 0.05). However, esthetic crown lengthening surgery resulted in significant improvements in patient satisfaction and oral health–related quality of life from baseline to three months of follow-up (*p* < 0.001). Photobiomodulation did not enhance primary wound healing or reduce postoperative pain following esthetic clinical crown lengthening with osteotomy. Nevertheless, the surgical procedure significantly improved patient satisfaction and quality of life over a three-month follow-up period.

## Background

Smile esthetics depend on the harmony among tooth shape and position, gingival levels, and lip position during smiling [1, 2]. Excessive gingival display (EGD), commonly referred to as a “gummy smile,” occurs when gingiva is overexposed during smiling or speaking, often leading patients to seek esthetic and functional treatment [1, 3]. Several etiological factors may contribute to EGD, including altered passive eruption (APE), gingival hyperplasia, and short clinical crowns, which may act independently or in combination [1, 4]. Clinically, EGD may result from abnormal dental development or gingival overgrowth characterized by connective tissue expansion and occasional cellular increase [5, 6].

Because of its esthetic impact, different surgical approaches have been proposed to achieve gingival symmetry and crown lengthening [7, 8]. Among them, clinical crown lengthening (CCL) is the most common treatment for excessive gingival display [9]. As described by recent literature, CCL is a technique aimed at exposing more tooth structure by apically displacing gingival and/or bone tissue for esthetic or reconstructive purposes [10, 11]. Beyond improving smile esthetics, CCL is widely indicated to correct premature eruption of the anatomical crown (altered passive eruption), reestablish the biologic width, and provide sufficient tooth structure for anterior restorations [10, 12, 13]. However, wound healing after CCL is relatively slow, often delaying restorative treatment that depends on prior periodontal plastic surgery [14]. In this context, low-level laser therapy (LLLT) has attracted attention for its photobiomodulatory potential to accelerate tissue repair and reduce postoperative pain [15].

LLLT, currently referred to as photobiomodulation (PBM), is based on the use of irradiation at specific wavelengths capable of modulating cellular behavior [16, 17]. This effect occurs through interactions with the mitochondrial respiratory chain or calcium channels, leading to increased metabolism and cell proliferation [18, 19]. Experimental studies have shown that PBM can stimulate fibroblasts, immune and epithelial cells, angiogenesis, growth factor release, and pain modulation, ultimately favoring wound healing [17, 20]. Its applications have been reported in temporomandibular disorders, paresthesia, orthodontic pain, dentin hypersensitivity, and periodontal wound healing [19, 21, 22].

Despite promising results, randomized clinical trials (RCTs) investigating photobiomodulation (PBM) show considerable heterogeneity in treatment protocols and a lack of standardization [23]. Variations are reported in key irradiation parameters, including wavelength (approximately 618–1064 nm), power output, energy density, irradiation time, and total energy delivered [24] In addition, differences in application techniques (contact versus non-contact modes, number and location of irradiation points), as well as treatment frequency and duration, have been described. In some studies, essential parameters such as spot size and energy dose are insufficiently reported, further limiting reproducibility and comparability across studies [24, 25]. Furthermore, the vast majority of existing RCTs have evaluated wound healing and pain solely in procedures characterized by healing by secondary intention, such as gingivectomies or palatal grafts, where outcomes are generally favorable for tissue repair and analgesia [7, 19, 21, 22, 26]. However, the scientific rationale for investigating primary intention healing lies in the fact that its cellular and molecular mechanisms differ from those involved in secondary intention healing. As secondary healing involves greater formation of granulation tissue and wound contraction, the modulatory effects of PBM cannot be directly extrapolated to primarily closed surgical flaps [27].

Additionally, esthetic CCL requires osteotomy, which introduces a more complex wound environment involving bone tissue. In this scenario, the biological need for PBM is justified by its capacity to increase mitochondrial membrane potential and ATP levels, triggering chemical signals that stimulate mesenchymal stem cells and the release of growth factors like TGF-b1, which are essential for bone and periodontal tissue repair [28]. To date, literature assessing the effects of PBM specifically following esthetic CCL with osteotomy remains limited and highly controversial. While some recent findings suggest that specific laser protocols may improve early hemostasis and reduce inflammation [23], other studies report that PBM has a strictly limited effect on postoperative outcomes, showing no significant reduction in overall pain intensity compared to controls [10].

To address these controversial findings and the scarcity of standardized protocols for surgical sites involving osteotomy, the aim of this study was to evaluate the efficacy of PBM on primary wound healing, postoperative pain, and other patient-centered outcomes after esthetic clinical crown lengthening surgery with osteotomy. The null hypothesis was that PBM would not influence wound healing, postoperative pain, or patient-reported outcomes compared with surgery alone.

## Materials and methods

This randomized, controlled, split-mouth, triple-blind, pilot clinical trial was conducted at the Department of Dentistry, Federal University of Rio Grande do Norte (UFRN), Brazil. The study was reviewed and approved by the Research Ethics Committee of the Federal University of Rio Grande do Norte (UFRN), Brazil (protocol no. 5.064.188), and was conducted in accordance with Resolution No. 466/12 of the Brazilian National Health Council. Written informed consent was obtained from all participants prior to enrollment in the study. This clinical trial was prospectively registered in the Brazilian Clinical Trials Registry (ReBEC) under the identifier RBR-9fbrcsy (registered on July 29, 2024).

Eligibility criteria included systemically healthy adults aged 18 years or older presenting excessive gingival display associated with APE or gingival zenith discrepancies in the anterior maxilla (minimum of six teeth) and clinical indication for esthetic crown lengthening with osteotomy. The indication for osteotomy was determined clinically based on periodontal probing to the alveolar crest, evaluation of gingival tissue proportions, and crown dimensions. Periodontal health was required, defined as bleeding on probing < 10%.

Exclusion criteria were: (1) cases requiring gingivectomy or gingivoplasty; (2) gummy smile associated with vertical maxillary excess or upper lip hypermobility; (3) drug-induced gingival overgrowth, esthetic restorations, orthodontic appliances, active periodontal disease, or reduced periodontium; (4) smoking, pregnancy, or lactation; (5) systemic conditions compromising healing; (6) allergy to study medications; and (7) failure to complete the laser sessions.

### Randomization and blinding procedures

Randomization was performed prior to surgery by an independent researcher using the Randomizer mobile application, which randomly assigned each hemiarch to either the control or test condition. Allocation concealment was maintained throughout the study. This investigation was conducted as a pilot randomized clinical trial designed to generate preliminary data and evaluate the feasibility of the proposed protocol; therefore, a formal a priori sample size calculation was not performed. A triple-blind design was adopted, in which patients, the surgeon responsible for the procedures, and the examiner performing the outcome assessments were blinded to group allocation. To ensure masking, both the patient and the operator wore protective glasses during all sessions. In the control hemiarch, sham laser irradiation was performed using the same application time and sweeping movements but with the device turned off, ensuring that no energy was delivered. Laser application was performed by a separate operator who was not involved in the surgical procedures or outcome evaluations. The control group received crown lengthening surgery alone, whereas the test group received surgery followed by photobiomodulation therapy.

### Examiner calibration

Prior to data collection, two blinded examiners were calibrated to ensure measurement reliability. The calibration process involved a review of all clinical parameters followed by duplicate assessments in five patients, with two measurements performed at a 30-minute interval. For quantitative variables, intra-examiner reliability was assessed using the intraclass correlation coefficient (ICC), yielding values of 0.853 for probing depth (PD) and 0.799 for keratinized mucosa width (KM). For categorical variables, agreement was evaluated using Cohen’s kappa coefficient (κ), resulting in κ = 0.859 for the modified Landry Healing Index and κ = 0.847 for the Pink Esthetic Score (PES).

### Outcome measures

The primary outcome of this study was primary wound healing following esthetic clinical crown lengthening surgery with osteotomy. Wound healing was assessed using a modified Landry Healing Index, scored from 1 (very poor) to 5 (excellent), based on standardized clinical photographs obtained at 4, 7, 11, 14, 45 days, and 3 months postoperatively.

Secondary outcomes included postoperative pain, esthetic outcomes, and patient-centered measures. Postoperative pain was recorded using a Numerical Visual Scale (NVS) at 8, 24, and 48 h, and from days 3 to 7 after surgery [29]. Esthetic outcomes were evaluated using the Pink Esthetic Score (PES) based on standardized intraoral and extraoral photographs obtained at baseline, immediately post-surgery, and during follow-up (Fürhauser et al., 2005).

Patient-centered outcomes included patient satisfaction, assessed using a visual numeric scale (1–5) at the same time points [29], and oral health-related quality of life, evaluated using the OHIP-14 questionnaire, which encompasses seven domains: functional limitation, physical pain, psychological discomfort, physical disability, psychological disability, social disability, and handicap [30].

Additionally, periodontal parameters, including bleeding on probing (BOP), probing depth (PD), and gingival phenotype, were recorded for clinical characterization purposes.

### Surgical protocol and PBM application

Preoperative care included oral hygiene reinforcement and, if necessary, scaling and polishing. Crown lengthening surgery was performed with a full-thickness flap and osteotomy in cases of altered passive eruption or malpositioned gingival zeniths. Standard antisepsis and local anesthesia with 2% articaine with epinephrine were applied. Trans-surgical probing guided tissue removal, followed by internal bevel and intrasulcular incisions, flap elevation, osteotomy, and flap repositioning with simple and vertical mattress sutures [31].

**Fig. 1:**
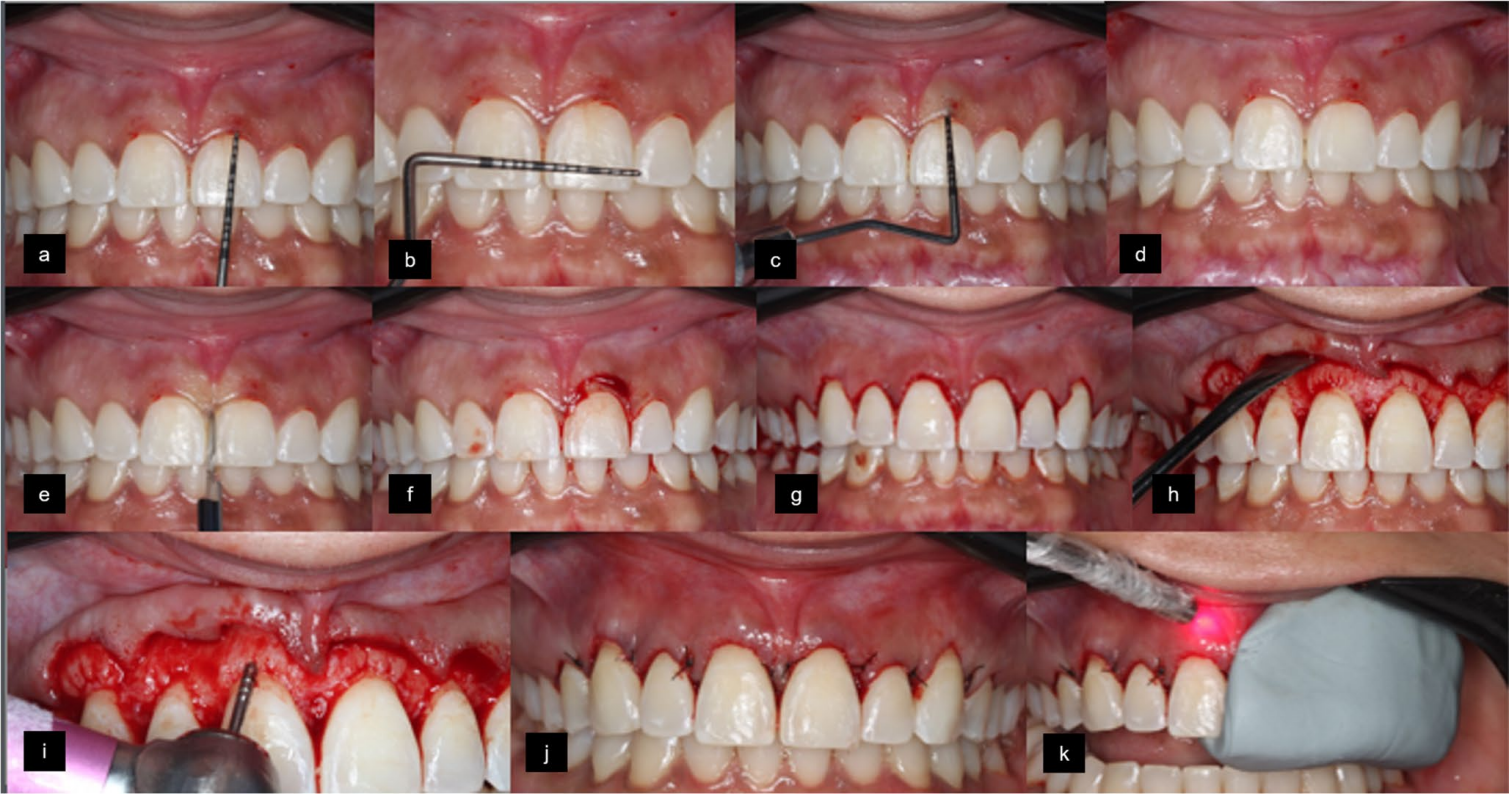
Step-by-step surgical protocol and PBM application. (**a**, **b**) Measurement of tooth height and width. (**c**) Probing to the alveolar crest to determine gingival tissue to be incised. (**d**) Identification of bleeding points. (**e**, **f**) Internal bevel incision. (**g**) Overview of all teeth in the surgical area with internal bevel. (**h**) Full-thickness flap. (**i**) Osteotomy to reestablish 3 mm distance between cemento-enamel junction and alveolar crest. (**j**) Simple interrupted sutures used for flap stabilization. (**k**) PBM application and placement of silicone barrier in the control hemiarch

Photobiomodulation (PBM) parameters were adapted from previous studies [7, 19]. In the test group (TG), a 660 nm diode laser (Therapy EC, DMC Equipamentos Ltda, São Carlos, SP, Brazil) was applied in continuous wave mode using a scanning technique, with an output power of 100mW, in a noncontact manner, for 5 min (Table 1). PBM was initiated immediately after surgery and repeated at 4, 7, 11, and 14 days postoperatively. In the control group (CG), patients underwent the surgical procedure followed by a sham laser application to maintain masking. A custom-made condensation silicone barrier (Zetaplus^®^, Zhermack) was placed over the contralateral hemiarch to prevent any unintended exposure of the control side to active irradiation [15] (Fig. 1).

### Surface area determination

**Fig. 2:**
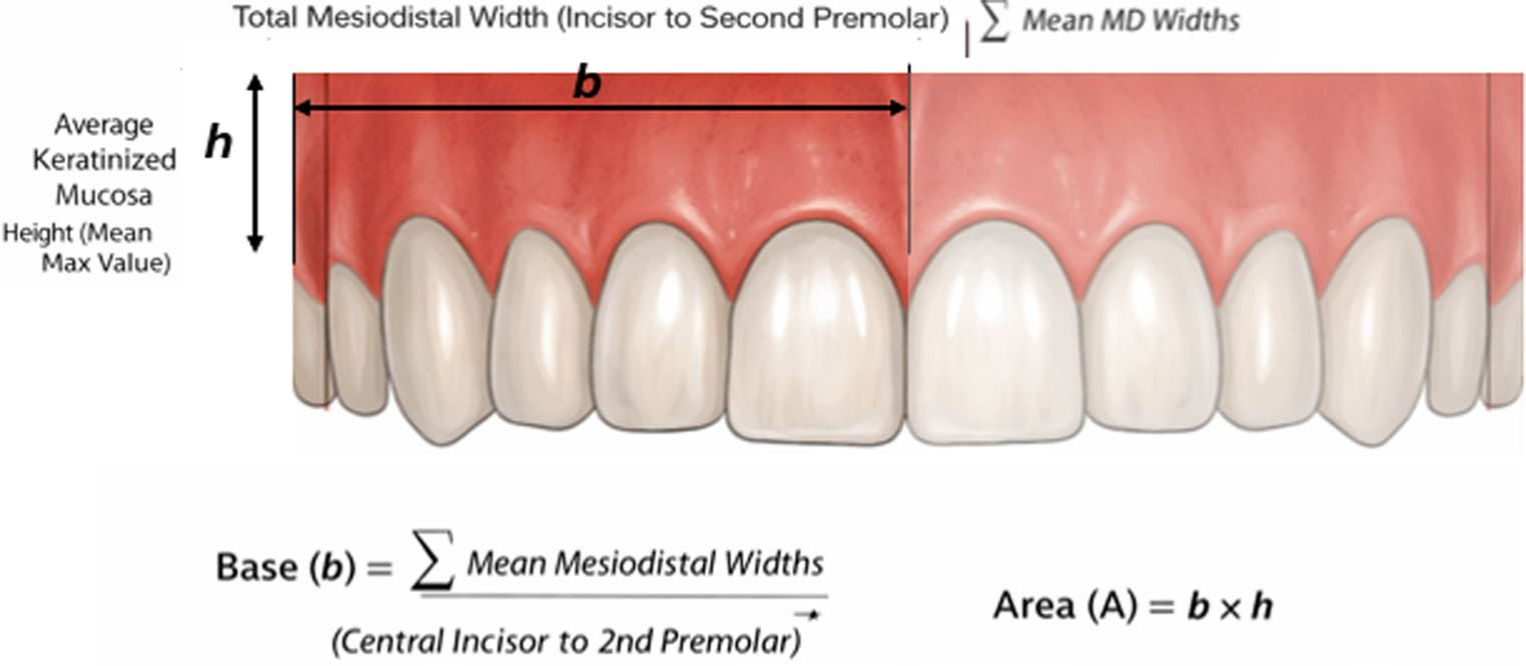
Laser irradiation area (split-mouth) approximated as a rectangle: base (b) = sum of mean mesiodistal widths (central incisor–second premolar); height (h) = mean maximum keratinized mucosa; A = b × h

The irradiated surface area was determined using a standardized geometric model based on established odontometric reference values and photobiomodulation dosimetry principles [32, 33]. Given the anatomical irregularity of gingival tissues in the postoperative setting, the treated area was approximated to a rectangular shape to allow reproducible and standardized calculation of energy density (J/cm²) [33, 34]. The horizontal dimension (base) was defined as the cumulative mean mesiodistal widths of the teeth included in the surgical field, extending from the central incisor to the second premolar. The vertical dimension (height) corresponded to a standardized representative value, calculated as the mean of the greatest measurements of keratinized mucosa recorded across the same dental elements [32, 34, 35]. This methodological approach was adopted to ensure reproducibility and standardization of the irradiated area, enabling accurate calculation of laser energy density. Such standardization is critical in photobiomodulation studies, in which the interaction between irradiated area, output power, and exposure time directly influences the biological response [33, 34] (Fig. 2).

Postoperative care included a cold liquid/soft diet for the first days, ice compresses for 72 h, chlorhexidine 0.12% mouth rinses starting 48 h after surgery and then used twice daily for 7 days, and analgesics (paracetamol 750 mg or dipyrone 500 mg, every 6 h as needed). Sutures were removed after 7 days.

### Statistical analysis

Data was entered into Microsoft Excel and analyzed using Jamovi^®^ (version 2.5.6.0). The Wilcoxon signed-rank test was used to evaluate changes in keratinized mucosa and smile classification. Patient satisfaction and oral health–related quality of life were analyzed using the Friedman test for repeated non-parametric measures, followed by Durbin–Conover post-hoc multiple comparisons to identify differences between time points. Postoperative pain, the modified Landry Healing Index, and the PES were analyzed using the Wilcoxon test for intragroup comparisons and the Mann–Whitney test for intergroup comparisons, with Bonferroni correction applied to adjust for multiple comparisons. The significance level was set at 5% (*p* < 0.05).

## Results

**Fig. 3:**
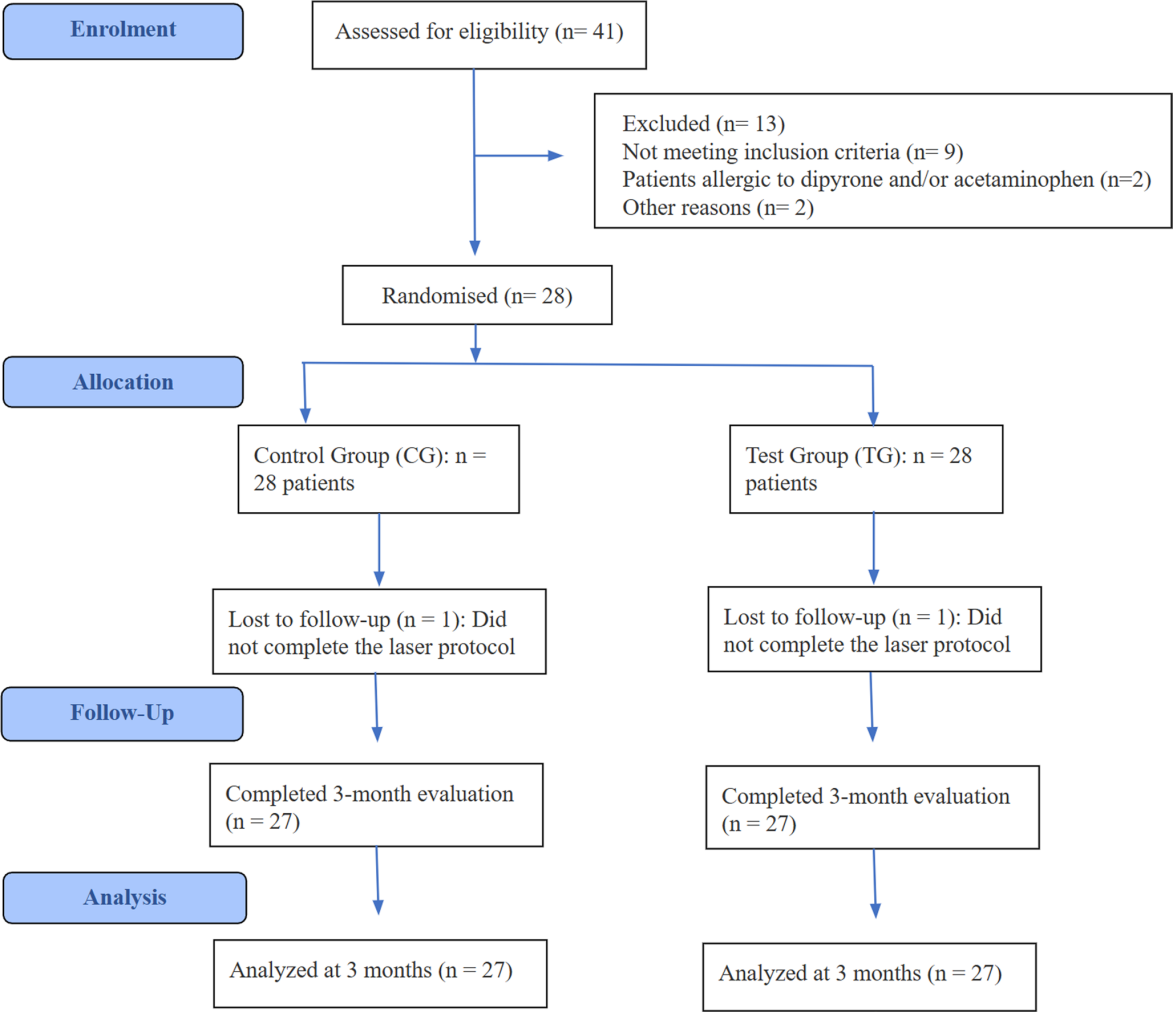
CONSORT flow diagram illustrating patient screening, allocation, follow-up, and analysis for the randomized, triple-blind, split-mouth clinical trial

A total of 41 patients were screened for eligibility between November 25, 2022, and May 13, 2024. Of these, 28 patients met the inclusion criteria, but one was lost during follow-up for not completing the full laser protocol. Thus, the final sample consisted of 27 patients, 24 females (88.9%) and 3 males (11.1%), with a mean age of 26.5 ± 5.57 (range: 19–43) and 25.3 ± 1.03 (range: 24–26), respectively (Table 1). Because of the split-mouth design, each patient contributed equally to the test group (GT, *n* = 27) and the control group (GC, *n* = 27), totaling 54 evaluated hemiarches. All surgeries extended over at least six teeth, ranging from canine-to-canine (13–23) or premolar-to-premolar (14–24 or 15–25), depending on the treatment plan, resulting in a total of 198 surgically treated teeth (54 central incisors, 54 lateral incisors, 54 canines, and 36 premolars) (Table 2). Seventeen patients presented a thin gingival phenotype (63.0%), whereas 10 presented a thick phenotype (37.0%). The study flow is detailed in the CONSORT diagram (Fig. 3).

**Table 1:** Device information and irradiation parameters

General settings	
Laser model	Therapy EC
Year of manufacture	2021
Manufacturer	DMC Equipamentos, São Carlos, São Paulo, Brasil
Wavelength	660 nm
Power	100mW
Emitter type	Diode
Tip area	0.028 cm^2^
Irradiation parameters	
Energy	30 J
Energy density	≈ 15 J/cm²
Exposure time	5 min
Application mode	Continuous and scanning mode
Irradiated area	2.04 cm²
Application technique	The laser tip was positioned perpendicular to the long axis of the tooth, in a non-contact mode, at a distance allowing simultaneous irradiation of the papillary region and keratinized mucosa.
Application frequency	Immediately postoperatively and repeated on days 4, 7, 11, and 14.

**Table 2:** Sample characterization according to age, gender, and gingival phenotype of individuals in the study groups

*n*	Gender				
	Male		Female		
27 (100%)	3 (11.11%)		24 (88.88%)		
*n*	**Age**				
	**Male ** **mean**		**Female ** **mean**		
27 (100%)	26.5 (19–43; ± 5.57 )		25.3 (24–26; ±1.03)		
*n*	**Phenotype**				
	**Thin**		**Thick**		
27 (100%)	17 (63.0%)		10 (37.0%)		
*n*	**Surgically treated teeth**				
	**Central incisors**	**Lateral incisors**		**Canine**	**Premolar**
198 (100%)	54 (27.3%)	54 (27.3%)		54 (27.3%)	36 (18.1%)

Clinical periodontal parameters, including BOP, PD, and KM, confirmed that all patients were periodontally healthy at baseline and remained so at the 3-month follow-up. According to the dichotomized thresholds (BOP < 10%, PD < 3 mm, KM > 2 mm), no patient developed periodontal disease during the study. 

Patient satisfaction and oral health–related quality of life showed a statistically significant improvement over time following esthetic crown lengthening surgery (*p* < 0.001). Post-hoc Durbin–Conover multiple comparisons revealed that these differences occurred between baseline and all subsequent evaluation time points (Table 3). From the immediate postoperative period up to the three-month follow-up, both outcomes demonstrated a linear pattern, with no statistically significant differences between consecutive time points, indicating stabilization after the initial postoperative improvement. For postoperative pain, intragroup analysis revealed significant reductions in pain levels over time in both groups (*p* < 0.001), but intergroup comparisons did not show statistically significant differences at any time point (Table 4).

**Table 3:** Influence of clinical crown lengthening surgery on patient satisfaction and oral health–related quality of life

PS – Patient satisfaction						
*N* %	*Baseline* Median (Q25–Q75)	Immediate postoperative Median (Q25–Q75)	14 days Median (Q25–Q75)	45 days Median (Q25–Q75)	3 months Median (Q25–Q75)	*p*
27 (100%)	3.00 (2.00–3.00) ^a^	5.00 (5.00–5.00) ^b^	5.00 (5.00–5.00) ^b, c^	5.00 (5.00–5.00) ^b, c,d^	5.00 (5.00–5.00) ^b, c,d, e^	< 0.001
OHIP-14 – Oral Health–Related Quality of Life						
27 (100%)	3.00 (2.00–5.00) ^a^	-	0.00 (0.00–0.00) ^b^	-	0.00 (0.00–0.00) ^b, c^	< 0.001

*p*-value obtained using the Friedman test for nonparametric repeated measures and post-hoc Durbin–Conover multiple comparisons test to identify at which evaluation time the statistical difference

**Table 4:** Intergroup and intragroup analysis of the photobiomodulatory effect of the laser in the test group and control group for postoperative pain at different evaluation times

	Postoperative pain		
Time point	Test group	Control group	*P* ^2^
8 h	2.00 (1.00–2.00)	2.00 (1.50–2.00)	0.52
24 h	1.00 (1.00–2.00)	1.00 (1.00–2.00)	0.50
48 h	1.00 (1.00–2.00)	1.00 (1.00–2.00)	0.78
3 days	1.00 (1.00–1.50)	1.00 (1.00–2.00)	0.51
4 days	1.00 (1.00–1.00)	1.00 (1.00–1.00)	0.97
5 days	1.00 (1.00–1.00)	1.00 (1.00–1.00)	0.69
6 days	1.00 (1.00–1.00)	1.00 (1.00–1.00)	0.16
7 days	1.00 (1.00–1.00)	1.00 (1.00–1.00)	0.33
*P* ^*1*^	**< 0.001**	**< 0.001**	

Different superscript letters indicate statistically significant differences within each group across evaluation periods, according to Friedman (p₁) and Wilcoxon (*p* < 0.05) tests. p₂ indicates intergroup differences (test vs. control) assessed with the Mann–Whitney test

Healing outcomes also improved progressively in both groups. The Landry healing index increased over time (*p* < 0.05), with no significant differences between groups (Table 5). Similarly, the PES showed progressive improvement in both groups (*p* < 0.05), but intergroup comparisons revealed no statistically significant differences (Table 6). Overall, photobiomodulation did not accelerate wound healing or reduce postoperative pain following esthetic crown lengthening; however, it exerted a positive impact on patient satisfaction and oral health–related quality of life after three months of follow-up.

**Table 5:** Intergroup and intragroup analysis of the photobiomodulatory effect of the laser in the test group and control group for Landry Index (LI) across evaluation times

	Landry Index		
Time point	LI- Test group	LI- Control group	*P* ^2^
4 days	3.00 (3.00–4.00) ^a^	3.00 (2.00–3.75) ^a^	0.37
7 days	4.00 (4.00–5.00) ^b^	4.00 (3.00–4.75) ^b^	0.14
11 days	4.00 (4.00–5.00) ^c^	4.00 (4.00–5.00) ^c^	0.61
14 days	5.00 (4.00–5.00) ^d^	5.00 (4.00–5.00) ^d^	0.71
45 days	5.00 (5.00–5.00) ^e^	5.00 (5.00–5.00) ^e^	0.31
3 months	5.00 (5.00–5.00) ^e, f^	5.00 (5.00–5.00) ^e, f^	0.57
*P* ^*1*^	**< 0.001**	**< 0.001**	

Different superscript letters indicate statistically significant differences within each group across evaluation periods, according to Friedman (p₁) and Wilcoxon (*p* < 0.05) tests. p₂ indicates intergroup differences (test vs. control) assessed with the Mann–Whitney test

**Table 6:** Intergroup and intragroup analysis of the photobiomodulatory effect of the laser in the test group (TG) and control group (CG) for Pink Esthetic Score (PES) across evaluation times

	Pink Esthetic Score		
Time point	PES- Test group	PES- Control group	*P* ^2^
4 days	1.00 (1.00–2.00) ^a^	1.00 (1.00–1.00) ^a^	0.54
7 days	2.00 (2.00–2.00) ^b^	2.00 (1.00–2.00) ^b^	0.07
11 days	2.00 (2.00–2.00) ^c^	2.00 (2.00–2.00) ^c^	0.69
14 days	2.00 (2.00–2.00) ^c, d^	2.00 (2.00–2.00) ^c, d^	0.32
*P* ^*1*^	**< 0.001**	**< 0.001**	

Different superscript letters indicate statistically significant differences within each group across evaluation periods, according to Friedman (p₁) and Wilcoxon (*p* < 0.05) tests. p₂ indicates intergroup differences (test vs. control) assessed with the Mann–Whitney test

## Discussion

The aim of this first triple-blind pilot randomized clinical trial was to evaluate the efficacy of photobiomodulation (PBM) on primary wound healing, postoperative pain, and patient-centered outcomes following esthetic clinical crown lengthening with osteotomy. The main findings indicate that PBM did not significantly improve primary wound healing, postoperative pain, or esthetic outcomes when compared with surgery alone. However, PBM significantly improved patient satisfaction and oral health–related quality of life at the three-month follow-up, representing the most clinically relevant outcome observed in this trial. The lack of significant effects on the primary outcome, tissue healing assessed by the modified Landry Healing Index [36], may be explained by the specific characteristics of the surgical procedure and the irradiation protocol used. In contrast to studies [21, 26, 37–39] evaluating secondary intention healing, full-thickness flap procedures involve deeper connective tissue remodeling, which may require different photobiomodulation parameters to achieve a measurable biological effect. In this context, the irradiation parameters used in the present study should be carefully interpreted from a biophysical perspective [40, 41]. Although the 660 nm wavelength primarily exerts superficial effects through the stimulation of cytochrome c oxidase and enhancement of cellular metabolism [42], the overall dosimetric profile, including output power (100 mW), energy density (≈ 15 J/cm²), and total irradiation time (5 min), may have influenced the biological response.

Photobiomodulation follows a biphasic dose–response pattern, described by the Arndt–Schulz law [40, 41], in which low doses stimulate biological activity, whereas higher doses may reduce or even inhibit cellular responses [43]. Thus, prolonged irradiation time and cumulative energy delivery may have shifted the response beyond the optimal therapeutic window, potentially resulting in a plateau or inhibitory effect [42, 43]. In addition, excessive energy deposition may increase the likelihood of photothermal effects, which are not the intended mechanism of action in low-level laser therapy and may interfere with cellular signaling pathways [40, 41]. These factors may partly explain the absence of significant effects on wound healing and postoperative pain observed in this study.

Furthermore, the effective dose delivered to the target tissue depends not only on energy density but also on irradiance, exposure time, and application technique [37]. In the present study, the sweeping (non-punctual) application may have resulted in a broader but less concentrated energy distribution, potentially diluting the therapeutic effect at specific target sites [37]. Additionally, the use of a red wavelength (660 nm) may have limited the depth of light penetration compared with near-infrared wavelengths (e.g., 810–980 nm), which are known to reach deeper tissues due to reduced absorption and scattering in biological structures [40]. This aspect is particularly relevant in full-thickness flap procedures, where deeper tissue layers play a central role in healing.

When compared with existing literature, these findings are consistent with studies reporting heterogeneous outcomes of PBM, largely attributed to variations in irradiation parameters and application protocols [7, 19, 21–23, 43–45]. While some studies [43] using near-infrared wavelengths have demonstrated improved healing and analgesic effects, particularly in deeper tissues, others have reported no significant differences, reinforcing the importance of parameter optimization. Experimental and clinical evidence suggests that near-infrared PBM may enhance tissue repair through angiogenesis, collagen synthesis, and modulation of inflammatory mediators, potentially resulting in improved wound healing and pain reduction [43]. Therefore, differences in wavelength selection, energy delivery, and application technique may contribute to the variability of clinical outcomes reported in the literature.

Regarding postoperative pain, both groups showed significant intragroup reductions over time, but no statistically significant differences were found between them. This aligns with earlier findings by Masse et al. (1993) [46], although other studies have demonstrated analgesic benefits of PBM after gingival grafts or flap surgeries [7, 47]. Variability in laser energy density, timing, total number of applications, and method of delivery may explain these inconsistencies. In the present protocol, PBM was applied in a sweeping motion rather than a punctual mode, which may have resulted in broader but less concentrated energy distribution, representing an additional methodological limitation.

Clinical periodontal parameters remained within healthy ranges throughout all evaluations, reflecting that all participants maintained adequate plaque control and periodontal stability, which is essential for predictable CCL outcomes [48]. Similarly, esthetic outcomes assessed through the Pink Esthetic Score did not differ significantly between groups, suggesting that PBM did not influence objective esthetic evaluations performed by calibrated professionals.

In contrast, patient-centered outcomes revealed a different pattern when analyzed over time. Patient satisfaction and oral health–related quality of life improved significantly throughout the follow-up period in both groups, reflecting the positive impact of esthetic clinical crown lengthening itself. These findings are particularly relevant in esthetic dentistry, where psychological and social dimensions strongly influence patient perception and treatment success [49]. The linear improvement observed after surgery suggests that changes in smile esthetics and gingival display play a central role in patients’ subjective assessment, regardless of adjunctive photobiomodulation.

This trial has some limitations, including the relatively small sample size and the absence of histological assessment, which could have provided deeper understanding of cellular response to PBM. Another limitation relates to the assessment of wound healing, which was performed using the modified Landry Healing Index based on standardized photographs. Although this index is widely used in periodontal studies and allows clinical evaluation of tissue color, bleeding, granulation tissue, and epithelialization, it remains a subjective method that depends on examiner interpretation. Moreover, the photographic evaluation may not fully capture subtle biological changes occurring in deeper tissues during the healing process. Future studies could incorporate more objective approaches, such as digital wound measurements, imaging-based analyses, or the evaluation of inflammatory and healing biomarkers, to provide a more comprehensive assessment of tissue repair.

Additionally, the split-mouth design may represent a methodological limitation in PBM studies, since local irradiation has been reported to induce systemic biological responses that could potentially influence non-irradiated sites. Experimental evidence suggests that PBM applied to the mandible may produce biological effects in distant tissues, indicating possible systemic signaling mechanisms [50]. Additional limitations include the choice of a red wavelength rather than infrared wavelengths and the sweeping (non-punctual) application technique, which may have increased light dispersion and potentially reduced the precision of energy delivery to deeper tissues. Nonetheless, these constraints do not invalidate the findings; the rigorous methodological design supports the robustness and internal validity of the results.

In summary, while PBM did not accelerate primary wound healing or reduce postoperative pain following esthetic crown lengthening with osteotomy, significant improvements in patient satisfaction and oral health–related quality of life were observed throughout the follow-up period after surgery. Future investigations should focus on optimizing PBM parameters and incorporating direct and objective assessments of inflammation and tissue repair, such as volumetric analyses, thermography, biochemical markers, or advanced imaging techniques, rather than relying solely on photographic evaluation, which may not fully capture subtle biological differences.

## Conclusions

Photobiomodulation did not accelerate primary intention tissue healing or reduce postoperative pain following esthetic clinical crown lengthening surgery. Improvements in patient satisfaction and oral health–related quality of life were observed over the three-month follow-up period.

## Data Availability

No datasets were generated or analysed during the current study.

## Declarations

### Artificial intelligence statement

Natural language processing was used exclusively for language refinement of a text entirely generated by the authors. All scientific content, data analysis, and interpretation were conceived and written by the authors.
